# Predictors of poor nutritional status among children aged 6–24 months in agricultural regions of Mali: a cross-sectional study

**DOI:** 10.1186/s40795-018-0225-z

**Published:** 2018-04-18

**Authors:** Caroline Makamto Sobgui, Leopold Kamedjie Fezeu, Fatou Diawara, Honafing Diarra, Victor Afari-Sefa, Abdou Tenkouano

**Affiliations:** 1 World Vegetable Center, West and Central Africa, PO Box 320, Bamako, Mali; 2 Université Paris 13, Equipe de Recherche en Epidémiologie Nutritionnelle (EREN), Centre de Recherche en Epidémiologie et Statistiques, Inserm (U1153), Inra (U1125), Cnam, COMUE Sorbonne Paris Cité, F-93017 Bobigny, France; 3 Agence National de Sécurité Alimentaire, Bamako, Mali; 4 West and Central Africa Council for Agricultural Research and Development, 7 Avenue Bourguiba, PO Box 48, Dakar, Senegal

**Keywords:** Stunting, Wasting, Underweight, Children, Mali

## Abstract

**Background:**

The right nutrition during the first 2 years of life can positively impact a child’s ability to develop, grow, and learn. Malnutrition remains a public health problem in Mali and little is known about the factors affecting the nutritional status of children. This study aims to assess the magnitude and the predictors of undernutrition in children aged 6–24 months in the poor rural regions of Mali.

**Methods:**

A community-based cross-sectional study was conducted in the villages in the Sikasso and Mopti regions in Mali from January to March 2016, comprising of 959 boys and 856 girls aged 6–24 months. A structured interviewer administered a questionnaire that was used to collect data from the mothers living in 1764 households. Anthropometric measurements were performed using standardized methods in order to identify the factors associated with children suffering from undernutrition (stunting and wasting). Bivariate and multivariate logistic regression analyses were conducted.

**Results:**

The results of our study indicated that 23.9 and 28.4% children were underweight and stunted; the prevalence of wasting was 13.9% using the W/H measurement and 16.5% with the MUAC. Overall, the presence of diarrhea in the past 2 weeks (*p* < 0.001), higher child age (*p* < 0.001), male sex (*p* < 0.001), households with the lowest household amenity score (*p* < 0.002), and households with a low dietary diversity score (*p* < 001) were significantly associated with chronic malnutrition. The factors significantly associated with acute malnutrition were male sex (*p* < 0.01), preterm birth (*p* < 0.03), lower child age (0.001), a high number of siblings (*p* < 0.03), and living in a household with more months of inadequate food provisioning (*p* < 0.03).

**Conclusion:**

Child undernutrition is a critical public health problem in the agricultural regions of Mali. Future efforts should be directed at addressing the food insecurity and at improving the yearlong household availability and accessibility of nutritious food, as well as taking diseases prevention into account.

## Background

Childhood malnutrition is a major public health problem in low-income countries. Approximately 30% of the total childhood mortality can be related to stunting or being underweight [1, 2]. Therefore, protecting children from malnutrition-inducing conditions during the first years of their life is critical for their future development [3–7]. Identifying the underlying causes of malnutrition is the first step to appreciate the scale and depth of the problem and to develop appropriate prevention strategies. A conceptual framework on the causes of malnutrition was developed as part of the United Nations Children’s Fund (UNICEF) Nutrition Strategy. This framework classifies the causes of malnutrition as immediate (child level: inadequate dietary intake, diseases like malaria, diarrhea, and acute pulmonary infections), underlying at the household level (insufficient access to food, inadequate maternal child care, poor water and sanitation quality, and inadequate health services) and underlying at the societal level (quality and quantity of the actual human, economic and organizational resources, environment, and technology). The presence of factors at one level influences other levels.

In Mali, malnutrition is a public health problem, as in most countries in sub-Saharan Africa [8]. It is one of the major causes of morbidity and mortality in children under 5 years of age. According to the 2012–2013 Mali Demographic Health Survey (http://www.dhsprogram.com/publications/publication-fa92-further-analysis.cfm, http://dhsprogram.com/publications/publication-mis24-mis-final-reports.cfm), the under-five-year-old mortality rate for the 5 years preceding the survey was 128‰ live births. In addition, among the children under 5 years old, 39% were stunted (Z-scores < − 2 SD height-for-age); 13% were wasted, or underweight for their height (Z-scores < − 2 SD weight-for-height); and 26% were underweight (Z-scores < − 2 SD weight-for-age). The Government of Mali and its technical and financial partners have developed and implemented a number of measures to improve health and nutritional status of the children in recent years, many of which emphasize local governance and leadership in pursuit of positive health outcomes. However, the identification of malnutrition predictors in order to develop target actions is also vital.

A number of authors in Sub-Saharan Africa, including Ethiopia [9–11], Kenya [12], Ghana [13], Tanzania [14], and Uganda [15, 16] have investigated the predictors of childhood undernutrition. Although the predictors reported varied according to the settings, they included food insecurity and food diversity [9, 11], child sex and age [10, 13, 15], childbirth weight [13], the parents’ educational level [10, 15], household socioeconomic status [13, 14], breastfeeding [17], water, sanitation, hygiene [12, 16], and child health among which diarrhea [10, 13, 17], fever, and coughing [16] were focused on. However, information about the predictors with respect to Mali is still missing in the literature. To the best of our knowledge, no such study has been conducted in Mali, nor using all the potential predictors of child malnutrition. Hence, there is a gap in the literature which this paper intends to fill by using a cross-sectional survey carried out in rural Mali to determine, among 6–24 months old children, which determinants among the immediate (child inadequate dietary intakes and health conditions) and underlying (household and family level: insufficient access to food, wealth, poor water, sanitation, and hygiene) factors are related to the nutrition status of children, defined using the following: wasting, stunting, and underweight.

## Methods

This study is part of the World Vegetable Centre project entitled “Deploying Improved Vegetable Technologies to Overcome Malnutrition and Poverty” funded by the United States Agency for International Development (USAID) in the regions of Sikasso and Mopti. This project is a community-based intervention, which aims to contribute to reduce malnutrition, especially of children and their mothers, through diet diversification by promoting the production and consumption of vegetables as affordable sources of essential vitamins and micronutrients.

### Study settings and target population

The rural regions of Sikasso and Mopti were selected as the study sites. The Sikasso inhabitants are mainly from the Bambara, Senufo, Miniankas, Fulani, and Samogo ethnic groups. Due to its favorable agricultural conditions, Sikasso is Mali’s biggest vegetable producer and considered one of the country’s most important bread baskets. The population of Mopti consists of the Dogon, Songhai, Bozo, Peulh, Bambara, and Tamaschek ethnic groups. The region is well watered in some parts and very dry in others, with agriculture and fishing being important economic activities. Mopti is a commercial crossroad between northern and southern Mali and its neighboring countries.

### Study design and study population

This study was a community-based cross-sectional study conducted between January and March 2016 using a multistage sampling approach. Three districts in Sikasso (Bougouni, Koutiala, and Sikasso) and two districts in Mopti (Koro and Bankass, which are in the dry zone) were purposively selected for the study and in each district, four municipalities were randomly selected. The final stage was the purposive selection of villages that had an existing health center. Additionally, the selected villages needed to be at least 50 km apart from each other.

A census was performed in all the villages to establish a sampling frame. The households were the Primary Sample Unit (PSU). Therefore, all the children in a household fulfilling the age criteria were potential participants. During the census, information on the households (marital status and educational level of parents, the total number of children, and the number of children between 6 and 24 months, and so on) was collected. A specific number was attributed to each household to facilitate the identification of those included in the sample collection. Other exclusion criteria were children with severe congenital malformations, children with serious illnesses or complications requiring hospitalization, as well as children whose parents declined to participate in the study. A random sampling per village, based on the households, was performed to constitute the final sample size of the study.

### Sample size calculation

The calculation of the sample size was based on the main objectives of the project, that is, improvement in the growth in status and reduced prevalence of diarrhea among the children of the intervention group compared with the control. The cluster effect was set at 1.5. We obtained a sample size of 2000 participants. This sample size was large enough to estimate the more prevalent malnutrition trait (stunting) at the national level at a 95% confidence interval and a 3% error precision.

### Data measurements

A pilot study was conducted to test the survey forms and procedures and these were adapted as necessary. The data was collected by three-member survey teams, comprising of at least one female worker. All enumerators were either medical doctors or nurses with survey experience and at least one enumerator in each team had participated in the census. They were all trained and certified for this study. The data were collected using structured questionnaires via face-to-face interviews and anthropometric measurements.

#### Outcome variable

The nutritional status of children aged 6–24 months, expressed as the prevalence of underweight, stunting, and wasting, was assessed using anthropometric variables such as height, Mid Upper Arm Circumference (MUAC), and weight. The weight was recorded on children wearing minimal clothing and bare feet using a standard calibrated weighing Uniscale (Seca®, Hamburg, Germany) in kilograms, to the nearest 0.1 kg. The height was taken using a stadiometer (Schorr®, UNICEF) in centimeters to the nearest 1 mm. The height and weight were taken twice and a difference of 0.1 cm in height and 100 g in weight was accepted as normal. The MUAC was measured thrice using non-stretchable tape on the left mid-upper arm to the nearest 1 mm.

Based on the recorded weight, the nutritional status was graded as per the 2006 WHO child growth standards using WHO Anthro version 3.2.2 software [18]. The children were considered stunted, wasted, or underweight if the height-for-age Z-score, the weight-for-age Z-score, or the weight-for-age Z-score was less than − 2 SDs (Standard Deviation) using the new WHO child growth standards, while those children with a score equal to or greater than − 2 SD were considered normal [18, 19]. During data processing, the exclusion criteria were applied to the anthropometric data of the children based on WHO recommendations to remove data that are most likely to be erroneous (HAZ and WAZ were excluded if the child value was <− 6.00 or > 6.00. The WHZ was excluded if the value was <− 4.00 or > 6.00). A MUAC below 12.5 cm indicated acute undernutrition and a value below 11.5 cm indicated severe acute undernutrition [20].

#### Explanatory variables

Three main types of questionnaires (for households, for mothers, and for children) were designed to record the data on any indicators of household socioeconomic and socio-demographic status, household food security, and care practices for children and their mothers.

The Household Food Insecurity Access Scale (HFIAS) was used to determine the household food insecurity including all nine generic questions that require recollection about the worry of food availability and accessibility in the previous months [21, 22]. The responses were summed to create a total score between 0 (the most food secure household) and 27 (the most food insecure household), which was determined using the Household Food Insecurity Access Prevalence (HFIAP) status indicator as a proxy of the household food insecurity prevalence [21]. Each household was then classified as either food secure, or mildly, moderately, or severely food insecure, based on the Food and Nutrition Technical Assistance (FANTA)'s recommended cut-offs. Information on durable assets (cupboard, hurricane lamp, radio, bicycle, boat, telephone, refrigerator, motorcycle, car, and so forth) and the materials of the dwelling structure were used to construct a relative index of the household wealth (asset) status using principal components analysis [23]. For each amenity available in the household, a score based on the Health/Nutrition/Population/Poverty Thematic Group of the World Bank for Mali was given and their sum was used as the household amenities score [24]. From the total household score, quartiles were computed and four wealth classes were defined, from the poorest (first quartile) to the richest (fourth quartile). The household dietary diversity scores (HDDS) were assessed using standard tools which collected information on the number of different food groups consumed over a given reference period among the 12 different food groups (cereals; roots and tubers; vegetables; fruits; meat, poultry, offal; eggs; fish and seafood; pulses/legumes/nuts; milk and milk products; oil/fat; sugar/honey; and spices/condiments/beverages) [25, 26]. The number of months during which the household was unable to meet its food needs during the last 12 months was collected using a structured questionnaire developed by FANTA [27]. Other household indicators included household possession of a latrine (available or not available), the presence of a fence around the household, the educational level of the household’s head, livestock, and the disposal of garbage.

The characteristics of the mother included the maternal age in years, educational level, current occupation, number of children, and vegetable intake. The educational status was measured according to the education levels in Mali: no formal education, having a primary level education, or secondary level and above.

The characteristics of the children included gender, the term of delivery (< 37 weeks or ≥ 37 weeks) and age in months; immunization against BCG and Penta 3 + Polio; deworming status in the past 6 months (yes or no); and a history of illness episodes. To assess childhood illnesses, the mothers were asked whether their children had been affected by diarrhea, fever, or coughs in the past 2 weeks. Diarrhea was defined as having three or more loose or watery stools in a 24-h period in the 2 weeks prior to the survey [28]. Assessment of feeding covered breastfeeding (yes or no) and dietary diversity of the children. The quality of complementary feeding was assessed using the individual dietary diversity score adapted for children, with 8 items instead of 12 as was the case for the household [26].

### Statistical analyses

Data were recorded using the Epi-Data® version 3.1 software and subjected to statistical analysis using the Stata® 11.1 software. Statistical procedures were adapted to the sampling methods (multi-stage random sampling with clusters being the primary sampling unit). For each participant included in the study, a weighting of the inverse multiple stage probability of being selected in the study was computed and the survey data analyses procedures of the Stata software were used.

Descriptive statistics were computed and the results were presented as means (95% confidence intervals) or medians (25th–75th percentiles) for the quantitative variables or as percentages for the qualitative variables. Comparison between the means or percentages was performed using a Student’s t-test or a chi-squared test, taking into account the complex sampling frame. Bivariate analyses were done for the four outcome variables: stunting, underweight, and wasting (using both WHZWHO and MUAC) separately. The independent variables with a *p*-value less than 0.20 for at least one of the nutritional parameters during bivariate analyses were selected as the candidates for multivariable analyses. Multivariable binary logistic regressions were fitted to identify the determinants of underweight, stunting, and wasting, separately. Associations between the dependent and independent variables were assessed using an Odds Ratio (OR) and a 95% confidence interval (CI). The tests were two-sided, and the statistical associations were declared significant if the *p*-values were less than 0.05.

### Ethical considerations

The protocol of the present study was approved by the Ethical Board of the University of Bamako, Faculty of Medicine, Pharmacy, and Odonto-stomatology of Mali (N°2016/44/CE/FMPOS). The objective of the study, the confidentiality, and the right to withdraw at any time without facing any consequences were explained to each participant in the local language at the time of recruitment. Written informed consent was obtained from each participant before any study enrollment. Every mother-infant pair meeting the inclusion criteria was considered for the study after obtaining the informed consent of the mother. Informed consent was acquired from a legal guardian for participants under 16 years of age at the time of the study. Participants with diarrhea, respiratory tract infections, and undernutrition were referred to health institutions and organizations working on nutrition.

## Results

### Characteristics of the study population

#### Households

Overall, 1897 households were invited to take part in the study, with a 95.3% response rate; 1808 households took part in the study. Due to missing values in anthropometric variables and main outcome variables, the analyses included 1764 households with 959 boys and 856 girls and their mothers. The mean household size was 6.7 (6.5–6.9) persons, in which the mean number of children aged 6–24 months was 1.05 (1.02–1.09). The mean age of the household’s head was 43 (39–46) years. Over 99.3% of the household heads were male, 74.9% were illiterate, and most were farmers (91.2%). Half of the households that took part in the study had no period of inadequate food provision while 11.8% of households had more than 4 months of inadequate food provision per year (Table 1). According to the computed score, 23.6% of households were food secure, and 41.0% had very low household food security access. For the latter, during the past month, some members of the household did not have enough food to meet their needs.

**Table 1:** The household, mother, and child characteristics of the study population

Characteristics	Mopti	Sikasso	P	Total
Household Characteristics				
N				1764
Months of inadequate household food provisioning, %			0.001	
0 month	28.6	70.1		50.7
1–3 months	56.2	21.3		37.5
4+ months	15.3	8.7		11.8
Household food insecurity access score categories, %			0.001	
Secure Food access	12.5	33.2		23.6
Mildly secure food access	8.7	7.5		8.1
Moderately insecure food access	12.5	35.1		23.3
Severely insecure food access	60.4	24.1		41.0
Household dietary diversity score, median (25th–75th percentiles)	4.8 (4.6–4.9)	5.0 (4.9–5.1)	0.001	4.9 (4.8–5.0)
Household amenities score, median (25th–75th percentiles)	0.47 (0.44–0.49)	0.54 (0.51–0.57)	0.001	0.50 (0.48–0.52)
Educational level of the father, %			0.001	
None	82.8	68.0		74.9
Literate	5.0	15.5		10.6
Primary or more	7.2	13.1		10.4
Other	5.0	3.3		4.1
Fence around the household, %	71.5	33.8	0.001	51.4
The household own livestock, %	37.8	32.9	0.04	35.2
The household owns a toilet facility, %	77.2	87.6	0.001	82.7
Trash dump/bin outside the household, %	77.2	87.6	0.001	82.7
Mothers’ Characteristics				
N				1764
Mean age (95% CI), years	27.2 (26.7–27.6)	27.6 (27.1–28.0)		27.3 (27.1–27.8)
Age classes, %			0.60	
< 24	35.8	36.9		36.4
25–34	46.6	44.2		45.3
35+	17.6	18.9		18.3
Occupation, %			0.001	
Housewife	85.7	61.9		72.8
Farmer	12.1	32.6		23.2
Other	2.2	5.5		4.0
Educational level, %			0.001	
None	93.4	81.4		86.9
Literate	5.6	17.3		12.0
Primary or more	1.0	1.3		1.1
Number of children alive, %			0.10	
1–2	35.5	36.0		33.9
3–4	31.4	31.2		31.3
5+	37.1	32.8		34.8
Vegetable intakes, days per week, %			0.001	
< 1	13.3	7.7		10.3
1–3	63.6	54.9		58.9
4+	23.1	37.3		30.8
Children’ Characteristics				
N				1815
Mean age (95% CI), months	13.9 (13.6–14.3)	14.7 (14.3–15.0)	0.004	14.3 (14.1–14.6)
Age group (months), %			0.05	
6–11	36.2	33.2		34.6
12–17	36.7	34.2		35.3
18–24	27.1	32.6		30.1
Sex of child, %			0.46	
Boy	52.0	53.8		53.0
Girl	48.0	46.2		47.0
Child born at term, %			0.001	
< 37 weeks	14.8	2.1		7.9
≥ 37 weeks	85.2	97.9		92.1
The child is still breastfed, %	90.2	87.0	0.04	88.5
Deworming therapy during the last 6 months, %	66.0	59.7	0.001	62.6
Diarrhea during the past 2 weeks, %	47.3	34.2	0.001	40.2
Fever during the last 2 weeks, %	53.1	44.5	0.001	48.5
Coughing during the last 2 weeks	35.3	28.7	0.001	31.7
IDDS class			0.71	
0–4	96.4	96.7		96.6
4+	3.6	3.3		3.4

The significance level was set at 0.05 for all the tests

*IDDS* Individual dietary diversity score

#### Mothers

The mean age of the mothers was 27.3 (27.1–27.8) years, with the age class 25–34 years being the most widely represented. Nearly all (99.5%) of the mothers were married with 72.8% being housewives; 86.9% were illiterate or had never attended school. One-third of the mothers had two children or less, one-third had between 3 and 4 children, and a third had 5 or more children alive.

#### Children

Nearly half (47%) of the children included in the study were girls. Their mean age was 14.3 (95% CI of the mean: 14.1–14.6) months. Two-thirds of children received both BCG and Penta 3 + HBV vaccines before they were 3 months old. About 40% of children had diarrhea in the last 15 days.

### Prevalence of underweight, stunting, and wasting

**Fig. 1:**
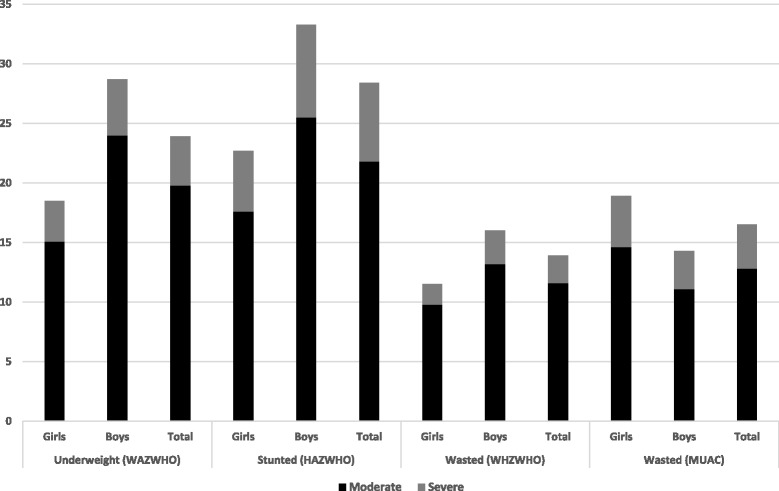
The prevalence (959 boys and 856 girls) of moderate and severe underweight, stunting, and wasting (using either WHZWHO or MUAC) among children aged 6–24 months in Mali. The data available for all the anthropometric characteristics of all the children

The mean (95% CI) of the WHZ, WAZ, HAZ (in SD), and MUAC (mm) of the children were − 1.20 (− 1.25; − 1.14), − 1.19 (− 1.25; − 1.13), − 0.79 (− 0.84; − 0.74), and 136.7 mm (136.1; − 137.2), respectively. The prevalence of underweight, stunting, and wasting among the study participants were 23.9% (21.9–26.0), 28.4% (26.3–30.6), and 13.9% (12.8–16.0) respectively. The prevalence of severe stunting, underweight, and wasting among the children were 4.1% (3.9–4.3), 6.6% (5.5–7.9), and 2.3% (1.7–3.2), respectively. The MUAC measurement also indicated that 16.5% (14.8–18.3) of the children were undernourished (< 12.5 cm), among which 3.7% (2.9–4.7) were severely undernourished (< 11.5 cm) (Fig. 1).

### Determinants of nutritional status

In the bivariate analyses, children living in wealthier households (*p* < 0.024), in households with a higher dietary diversity score (*p* < 0.03), or with adequate access to food provisioning (*p* < 0.03) had less prevalence of stunting (Table 2). Furthermore, households with livestock (*p* < 0.003), storing garbage in a trash dump/can outside the household (*p* < 0.04), or not suffering from inadequate food provision (*p* < 0.002) experienced the lowest prevalence of wasting. The characteristics of children associated with a higher prevalence of stunting (Table 3) included being male (*p* < 0.001), being older (*p* < 0.001), being breastfed (*p* = 0.01), and being reported as having diarrhea (*p* = 0.02). Only child age (*p* < 0.001), male sex (*p* = 0.009), term of delivery (*p* = 0.009), and reports of diarrhea during the past 2 weeks (*p* = 0.05) were associated with wasting. There was no relationship between the mothers’ characteristics and the nutritional status of the children (Table 4).

**Table 2:** The bivariate associations between the household characteristics and the child’s nutritional status

Household Characteristics	Underweight (%) WAZWHO	Stunted (%) HAZWHO	Wasted (%) WHZWHO	Wasted (%) MUAC < 115 cm
N	1815	1815	1815	1815
Months of inadequate household food provision				
0 months	22.6	28.4	13.8	16.3
1–3 months	23.0	26.7	13.6	14.0
4+ months	31.5	32.4	17.1	26.2
P trend	0.03	0.29	0.42	0.002
Household food insecurity score categories				
Secure Food access	20.3	27.0	14.0	18.7
Mildly secure food access	22.9	25.8	13.3	19.3
Moderately insecure food access	27.2	30.9	13.8	13.5
Severely insecure food access	23.7	27.7	14.5	17.0
P trend	0.16	0.45	0.80	0.16
Household dietary diversity score tertiles				
First	26.6	31.3	15.7	18.4
Second	21.5	26.8	14.8	15.6
Third	22.3	25.8	12.1	14.6
P trend	0.06	0.03	0.07	0.07
Household amenities score				
First quartile	27.0	34.8	14.3	19.5
Second quartile	25.2	27.4	13.1	16.0
Third quartile	21.5	26.7	14.9	15.6
Forth quartile	21.2	23.6	14.2	15.1
P trend	0.024	0.001	0.84	0.28
Educational level of the father				
None	24.2	28.0	15.1	17.0
Literate	23.2	25.0	9.9	14.1
Primary or more	22.9	32.0	11.0	14.8
Other	21.2	29.9	13.8	21.5
P trend	0.52	0.54	0.19	0.51
Fence around the household				
Yes	21.3	27.3	13.6	17.3
No	26.5	29.2	14.7	15.9
P	0.01	0.39	0.51	0.40
The household owns livestock				
Yes	20.7	29.2	10.8	15.2
No	25.5	27.6	15.9	17.4
P	0.03	0.49	0.003	0.24
The household owns a toilet facility				
Yes	23.8	28.4	14.4	17.0
No	23.7	27.6	12.8	15.0
P	0.97	0.78	0.46	0.39
Garbage in a trash dump outside the household				
Yes	16.7	26.0	8.0	18.6
No	24.4	28.4	14.6	16.5
P	0.058	0.58	0.04	0.54
Region				
Sikasso	25.4	31.5	12.3	17.0
Mopti	22.1	24.7	16.7	15.9
P	0.10	0.001	0.001	0.53

Data are the prevalence of each nutritional status according to the households’ characteristics classes

The significance level was set at 0.05 for all the tests

**Table 3:** The bivariate associations between the mothers’ characteristics and the child’s nutritional status

Characteristics	Underweight (%) WAZWHO	Stunted (%) HAZWHO	Wasted (%) WHZWHO	Wasted (%) MUAC < 115 cm
N	1815	1815	1815	1815
Age class, years				
< 24	23.6	28.7	13.9	16.4
25–34	24.2	26.8	14.8	15.5
35+	23.6	31.7	14.2	19.1
P trend	0.96	0.26	0.88	0.34
Occupation				
Housewife	24.1	27.6	14.1	16.1
Farmer	22.4	30.4	14.3	17.8
Other	28.1	31.8	18.1	15.2
P	0.57	0.47	0.66	0.71
Educational level				
None	24.5	28.7	15.0	16.8
Literate	20.3	25.6	9.5	15.5
Primary or more	17.7	30.4	11.9	0.0
P	0.34	0.67	0.13	0.13
Number of children alive				
1–2	25.2	30.2	14.2	14.9
3–4	22.5	25.0	14.4	15.4
5+	23.9	30.0	14.4	19.1
P	0.57	0.10	0.98	0.11
Vegetable intakes, days per week				
< 1	28.3	28.7	17.4	16.1
1–3	22.6	27.1	14.6	15.9
4+	25.3	31.1	12.9	17.0
P	0.18	0.27	0.30	0.86

Data are the prevalence of each nutritional status according to mothers’ characteristics classes

The significance level was set at 0.05 for all the tests

**Table 4:** The bivariate associations between the children’s characteristics and children’s nutritional status

Children Characteristics	Underweight (%) WAZWHO	Stunted (%) HAZWHO	Wasted (%) WHZWHO	Wasted (%) MUAC < 115 cm
N	1815	1815	1815	1815
Age group (months)				
6–11	21.3	17.8	14.1	20.8
12–17	26.6	28.8	18.4	17.8
18–24	23.7	40.1	9.7	9.9
P trend	0.09	0.001	0.001	0.001
Sex of child				
Boy	28.7	33.4	16.5	14.3
Girl	18.5	22.7	12.0	18.9
P	0.001	0.001	0.007	0.009
Child born at term				
< 37 weeks	26.9	22.2	21.4	24.8
≥ 37 weeks	23.6	28.9	13.7	15.8
P	0.37	0.08	0.009	0.003
The child is still breastfed				
No	25.3	27.3	12.4	13.5
Yes	23.5	36.0	14.6	16.9
P	0.61	0.01	0.42	0.22
Deworming therapy during the last 6 months				
Yes	24.4	30.2	14.8	16.7
No	23.1	25.5	13.8	16.4
Don’t know	21.8	22.1	10.7	12.0
P	0.78	0.057	0.66	0.67
Diarrhea during the past 2 weeks				
Yes	27.0	31.9	16.5	18.3
No	21.6	26.1	12.8	15.1
P	0.01	0.02	0.05	0.02
Fever during the last 2 weeks				
Yes	26.5	29.1	15.8	18.0
No	21.5	27.7	12.9	15.1
P	0.01	0.54	0.08	0.11
Coughing during the last 2 weeks				
Yes	26.1	29.8	15.0	16.5
No	22.8	27.7	13.9	16.4
P	0.30	0.65	0.07	0.85
Individual Dietary Diversity Score class				
0–4	23.9	28.1	14.4	16.4
4+	22.9	34.8	13.3	18.9
P	0.86	0.26	0.82	0.60

Data are the prevalence of the nutritional status according to the children’s characteristics classes

The significance level was set at 0.05 for all the tests

In multivariable analyses (Table 5), the presence of diarrhea in the past 2 weeks (*p* < 0.001), children being older (*p* < 0.001), being male (*p* < 0.001), households having the lowest household amenity score (*p* < 0.002), or households having the lowest dietary diversity score (*p* < 001) were associated with the highest prevalence of stunting. On the other hand, children born during the preterm (*p* < 0.03), being younger (*p* < 0.001), having a higher number of siblings (*p* < 0.03), and living in a household with more months of inadequate food provision (*p* < 0.03) were associated with a higher prevalence of wasting.

**Table 5:** The multivariate (odds ratios and 95% confidence intervals) associations between the household, maternal, and child characteristics and the nutritional status of the children

Household Characteristics	Underweight WAZWHO	Stunted HAZWHO	Wasted WHZWHO	Wasted MUAC < 115 cm
N	1815	1815	1815	1815
Months of inadequate household food provision				
0 months	1.00	1.00	1.00	1.00
1–3 months	1.06 (0.80–1.41)	0.97 (0.74–1.27)	0.83 (0.60–1.16)	0.91 (0.65–1.26)
4+ months	1.50 (1.03–2.18)	1.25 (0.86–1.84)	1.02 (0.66–1.60)	1.80 (1.20–2.71)
P trend	0.06	0.38	0.80	0.03
Household food insecurity access score categories				
Food secure access	1.00	1.00	1.00	1.00
Mildly food secure access	1.12 (0.68–1.84)	1.06 (0.65–1.71)	0.71 (0.39–1.28)	1.04 (0.62–1.75)
Moderately food insecure	1.27 (0.89–1.81)	1.16 (0.83–1.65)	0.77 (0.50–1.16)	0.67 (0.44–1.01)
Severely food insecure access	1.08 (0.75–1.56)	1.12 (0.79–1.58)	0.68 (0.45–1.02)	0.85 (0.58–1.23)
P trend	0.61	0.47	0.08	0.21
Household dietary diversity score tertiles				
First	1.00	1.00	1.00	1.00
Second	0.82 (0.61–1.08)	0.80 (0.60–1.05)	0.97 (0.70–1.35)	0.92 (0.67–1.26)
Third	0.84 (0.63–1.11)	0.71 (0.53–0.94)	0.71 (0.50–1.02)	0.83 (0.59–1.16)
P trend	0.18	0.01	0.07	0.27
Household amenities score quartiles				
First quartile	1.00	1.00	1.00	1.00
Second quartile	0.89 (0.65–1.21)	0.67 (0.50–0.92)	0.97 (0.66–1.44)	0.86 (0.60–1.24)
Third quartile	0.77 (0.55–1.07)	0.68 (0.50–0.95)	1.09 (0.73–1.62)	0.81 (0.55–1.19)
Forth quartile	0.81 (0.58–1.13)	0.52 (0.37–0.73)	1.18 (0.78–1.81)	0.85 (0.59–1.23)
P trend	0.15	0.002	0.37	0.34
Fence around the household				
Yes	1.00	1.00	1.00	1.00
No	1.13 (0.87–1.46)	0.94 (0.73–1.21)	1.20 (0.88–1.64)	0.76 (0.56–1.02)
P	0.35	0.62	0.24	0.06
The household owns livestock				
Yes	1.00	1.00	1.00	1.00
No	0.93 (0.71–1.22)	1.27 (0.98–1.64)	0.70 (0.51–0.96)	0.88 (0.65–1.20)
P	0.60	0.06	0.03	0.43
Garbage in a trash dump outside the household				
Yes	1.00	1.00	1.00	1.00
No	1.44 (0.84–2.45)	1.13 (0.69–1.86)	1.94 (0.98–3.87)	0.83 (0.51–1.36)
P	0.18	0.62	0.06	0.08
Region				
Mopti	1.00	1.00	1.00	1.00
Sikasso	1.4 (0.91–1.69)	1.46 (1.08–1.97)	0.64 (0.45–0.90)	1.37 (0.96–1.96)
P	0.17	0.01	0.01	0.08
Number of children alive				
1–2	1.00	1.00	1.00	1.00
3–4	0.88 (0.66–1.17)	0.75 (0.57–0.99)	0.99 (0.69–1.42)	1.03 (0.74–1.45)
5+	0.94 (0.71–1.25)	0.90 (0.68–1.18)	1.02 (0.73–1.43)	1.43 (1.03–1.97)
P trend	0.69	0.13	0.89	0.03
Age class of children (months)				
6–11	1.00	1.00	1.00	1.00
12–17	1.34 (1.02–1.77)	2.00 (1.50–2.66)	1.37 (1.00–1.87)	0.78 (0.58–1.06)
18–24	1.20 (0.87–1.66)	3.61 (2.63–4.95)	0.64 (0.41–0.99)	0.37 (0.24–0.57)
P trend	0.19	0.001	0.10	0.001
Sex of child				
Girl	1.00	1.00	1.00	1.00
Boy	1.78 (1.40–2.25)	1.73 (1.37–2.17)	1.46 (1.10–1.94)	0.71 (0.55–0.94)
P	0.001	0.001	0.008	0.015
Child born at term				
< 37 weeks	1.00	1.00	1.00	1.00
≥ 37 weeks	0.77 (0.51–1.15)	1.32 (0.84–2.08)	0.74 (0.47–1.15)	0.62 (0.40–0.95)
P	0.20	0.22	0.18	0.03
The child is still breastfed				
Yes	1.00	1.00	1.00	1.00
No	1.02 (0.68–1.53)	1.13 (0.78–1.63)	0.92 (0.54–1.58)	0.74 (0.45–1.23)
P	0.93	0.53	0.76	0.24
Deworming therapy during the last 6 months				
Yes	1.00	1.00	1.00	1.00
No	0.92 (0.71–1.19)	0.82 (0.64–1.05)	0.88 (0.64–1.21)	0.90 (0.67–1.22)
P	0.42	0.12	0.28	0.054
Diarrhea during the past 2 weeks				
Yes	1.00	1.00	1.00	1.00
No	0.88 (0.66–1.18)	0.64 (0.49–0.85)	0.83 (0.59–1.15)	0.84 (0.61–1.15)
P	0.40	0.002	0.26	0.27

Each model was further adjusted for the presence or absence of fever or coughs during the past two weeks, and the educational level of mothers

Data are odds ratios and 95% confidence intervals

The significance level was set at 0.05 for all the tests

## Discussion

This study aimed to assess the magnitude and determinants of the nutritional status of 6–24-month-year-old children in a rural area of Mali where agriculture is the main source of livelihood. Overall, 23.9% of the children were underweight, 28.3% were stunted, and 13.9% were wasted. According to the WHO criteria, the prevalence of underweight was “high”, the prevalence of stunting was “medium”, and the prevalence of wasting indicated a “serious problem”. Our findings confirm those obtained during the 2014 national DHS (http://www.dhsprogram.com/publications/publication-fa92-further-analysis.cfm, http://dhsprogram.com/publications/publication-mis24-mis-final-reports.cfm).

Children living in food insecure and very poor households had a higher likelihood to develop wasting and stunting than those living in food secure and wealthier households. Models developed to study food security and infant nutrition hypothesized that food insecurity leads to a decrease in the consumption of foods and nutrients rich in energy, thus, increasing the risk of children becoming ill [29–31]. Some authors [1, 5] have conducted studies among populations living in underprivileged areas and have reported rare dietary diversification and consumption of the same types of foods during most meals in many households. The fact that higher food security is associated with better child nutrition implies that poor households are unable to achieve their daily dietary needs. A household has optimal food security if all its members have durable and safe food of sufficient quality and quantity to ensure adequate intake and a healthy living [31]. In rural areas in Africa, household food is mainly based on harvesting as a means of livelihood. For this reason, their food security depends on access to agricultural land and the quality of agricultural production systems to meet sufficient domestic needs. Resources available to a household have to be enough to cover all the needs for the whole calendar year. The capability of the household to meet all the nutritional needs of its members during this period is affected by many parameters including incidents requiring additional income, soil fertility, and lack of manpower. For these reasons, resource-limited households have few chances to constitute enough stocks of food or to develop alternatives that would be used in times of hardness [32]. Therefore, increasing yields and production combined with developing storage techniques to ensure year-round food availability could help to alleviate the burden of child malnutrition.

We found that reporting episodes of diarrhea occurrence within the 2 weeks preceding the survey was an independent risk factor for stunting. The results of this study were in agreement with those studies conducted in other developing countries [3, 15, 33]. Infections are major risk factors for undernutrition. They are responsible for increased nutritional needs, higher energy expenditure, lower appetite, and nutrient losses due to vomiting and diarrhea. All this creates in addition to undernutrition as a disruption of the metabolic balance [4, 29]. The interaction between inadequate dietary intake and gastroenteritis tends to create a vicious cycle: A malnourished child will have lower disease resistance; for this reason, he will fall sick more often and, as a result, his malnutrition will worsen. Children entering this cycle of malnutrition-infection are less likely to be able to survive as one state feeds the other.

The probability of both stunting and wasting was higher among boys compared to girls. These results confirm the findings of other authors in Uganda and Ghana [15, 33, 34]. A possible explanation is that boys are more susceptible to environmental stress than girls. Therefore, they have a higher likelihood to display the consequences of stunting. This is particularly true in stressful conditions [15, 17, 34].

Our results, which are confirmed by earlier studies [35–37], also highlighted the fact that children born to mothers who gave birth to five or more children had a higher likelihood of being undernourished than children who were born to mothers with fewer children. It seems obvious that families with a large number of children are more vulnerable to economic constraints resulting from the need to fulfill the nutritional needs of the household. These families are therefore more likely to suffer from a poor nutritional status. Inadequate allocation of household resources among that many children can lead to the low nutritional status. In addition, families with more children generally spend less time caring for each child [35].

Maternal characteristics did not exhibit enough variability in our study population. For example, 1.1% of mothers attended more than primary school, 73% were housewives, and more than 80% were aged under 34 years. This could explain why these potential predictors were not associated with child malnutrition.

The limitations of this study include its cross-sectional design, which makes it difficult to examine potential temporal relationships and causality. A memory recall bias cannot be ruled out, for example, with regard to the child’s history of illness and breastfeeding behavior. Data were not collected on parasitic infections, birth weight, and daily caloric intake. These variables are potential confounders and their absence can lead to an erroneous interpretation of our results. A parasitic infestation can have an impact on the child’s nutrition and growth, altering the intestinal absorption of fats and nutrients [1, 36]. Moreover, information on the child’s birth weight, which is strongly associated with the child’s size, was not taken into account due to the high number of missing values. The exact daily caloric intake was not ascertained. Household wealth was assessed using indirect measures (household amenities and asset ownership) due to the inability to collect accurate information on household income and expenditure, which are better measures of household wealth.

## Conclusion

The results of this study showed that food insecurity and household poverty are major determinants of child malnutrition. Food insecurity is a public health problem and should be considered and managed as a social determinant of health. Policymakers should develop and implement social protection policies in Mali, in order to contribute to the reduction of the high rates of child malnutrition in Sikasso and Mopti. In addition, it is imperative that specific efforts be made to combat malnutrition in children. The policies put in place should increase the availability and sustainability of household food supply. They must also take into account possible diseases, which can counteract improvements in food security. Future efforts should maximize the capacity of families living in very poor and food insecure areas to provide their children with a more diverse diet throughout their childhood. Our findings point to the need to promote age-appropriate feeding practices in order to prevent stunting and increase the chances of recovery in children with stunted growth.

## Abbreviations

BCG vaccine: Bacillus Calmette–Guérin vaccine
CI: Confidence interval
FANTA: Food and Nutrition Technical Assistance
GRS: Growth reference standards
HAZ: Height-for-Age
HBV: Hepatitis B vaccine
HDDS: Household dietary diversity score
HFIAP: Household Food Insecurity Access Prevalence
HFIAS: Household Food Insecurity Access Scale
MUAC: Mid upper arm circumference
OR: Odd ratio
PSU: Primary sample unit
SD: Standard deviation
UNICEF: United Nations Children’s Fund
USAID: United States Agency for International Development
WASH: Water, sanitation, and hygiene
WAZ: Weight-for-age Z score
WHO: World Health Organization
WHZ: Weight-for-height
WorldVeg: World Vegetable Center
